# Lonely in the City–Sociodemographic Status and Somatic Morbidities as Predictors of Loneliness and Depression among Seniors–Preliminary Results

**DOI:** 10.3390/ijerph18147213

**Published:** 2021-07-06

**Authors:** Kasper Sipowicz, Marlena Podlecka, Łukasz Mokros, Tadeusz Pietras

**Affiliations:** 1 Department of Interdisciplinary Disability Studies, The Maria Grzegorzewska University in Warsaw, Szczesliwicka 40, 02-353 Warsaw, Poland; ksipowicz@aps.edu.pl; 2 Institute of Psychiatry and Neurology in Warsaw, Department of Neuroses, Personality Disorders and Eating Disorders, Sobieskiego 9, 02-957 Warsaw, Poland; mpodlecka@ipin.edu.pl; 3 Department of Clinical Pharmacology, Medical University of Łódź, Kopcinskiego 22, 90-153 Łódź, Poland; 4 Institute of Psychiatry and Neurology in Warsaw, Second Department of Psychiatry, Sobieskiego 9, 02-957 Warsaw, Poland; tpietras@ipin.edu.pl

**Keywords:** depression, psychogeriatry, morbidity, social support, cross-sectional study, linear regression

## Abstract

Up to a third of the population of older adults has been estimated to suffer from feelings of loneliness, which is considered a risk factor of depression. The aim of this paper is to compare the perceived level of loneliness and depression in seniors living in the country and in the cities and assess somatic morbidity and sociodemographic status as predictors of loneliness and depressiveness. *n* = 92 older adults in primary care units filled out a set of questionnaires: authors’ survey on sociodemographic data and morbidities, Beck Depression Inventory II (BDI, to measure depressiveness) and De Jong Gierveld Loneliness Scale (DJGLS, to assess loneliness). There was a strong, positive and statistically significant correlation between the BDI and DJGLS scores (R = 0.855, *p* < 0.001). City residents had on average higher BDI and DJGLS scores. Linear regression models were constructed to predict BDI and DJGLS scores. The set of statistically significant predictors were similar for BDI and DJGLS. Sociodemographic status and somatic morbidities accounted for around 90% of variance of depressiveness and loneliness scores in the studied group. Living alone was found to be the strongest relative predictor of both loneliness and depressiveness in the studied sample of the older adults. Our current results suggest that there might be a need to improve social support in the late adulthood as an intervention to diminish the sense of loneliness and depressiveness.

## 1. Introduction

Loneliness may be considered an attribute of late adulthood caused by a combination of events associated with that stage of life [1]. It is assumed that the leading category characterizing that period is the concept of “loss” (costs of aging), which takes the form of loss of health and physical attractiveness, loss of close persons, loss of socio-economic status, loss of the sense of prestige and usefulness, and a feeling of impending death. Such critical events characteristic of the period of aging, or, in fact, the difficulty in coping with the demands posed by them, can contribute to an increase in feelings of loneliness and the onset of symptoms of depression [1]. The departure of children from home, the loss of a life partner, the death of many friends, and the deterioration of health may cause sadness and “existential vacuum” in many individuals. The loneliness of senior citizens may be further exacerbated by economic poverty [2].

Cacciopo et al. define loneliness as a discrepancy between the preferred and actually available social relations [3]. According to Jenny de Jong Gierveld, loneliness can be defined as a situation in which an individual experiences an unpleasant and unacceptable quality, or lack of interpersonal relationships. Deprivation in this regard can be due to both insufficient number of interactions and incomplete sense of intimacy [4]. Loneliness should be considered as a kind of subjective sense of the individual, which consists of an individual perception, experience and evaluation of one’s own social interactions. This concept should not be equated with the objectively observed extent and quality of the subject’s network of interpersonal relations. Thus, people with few contacts experience social isolation, but this is not necessarily equivalent to loneliness, and vice versa [5]. The relationship between an objective indicator of social isolation and a subjective experience of loneliness is affected by the factors such as: characteristics of the relationships available, currently unavailable and no longer available; the significance of lost relationships; the time perspective; the perceived possibility of qualitative or quantitative improvement of the social relation networks; the personal characteristics (shyness, social competence, assertiveness); and self-conception [4].

Depression, on the other hand, is defined according to the DSM-5 criteria. However, it should be emphasized that the characteristic clinical picture of this disorder in older adults is qualitatively different from that of the general population. There is a clear tendency to cognitive impairment, psychomotor retardation or agitation, psychosis, high levels of anxiety, somatization, and hypochondria [6]. Therefore, specific depression subtypes specific to late adulthood have been identified: “depression without sadness”, or “depletion syndrome”, as well as “depression-executive dysfunction syndrome” [7].

Up to a third of the population of older adults has been estimated to suffer from the feelings of loneliness [8]. A 2009 meta-analysis shows that 20–30% of 65–79-year-olds and up to 40–50% of seniors over 80 years of age suffer from loneliness [9]. According to a recent report prepared by the National Academies of Sciences, Engineering, and Medicine, more than a third of the population above 45 years of age feels lonely, while nearly a quarter of over-65-year-olds experiences social isolation [10].

Despite such a catastrophic vision of senior loneliness, it is reported that mood disorders in this group of people are not more common than in the young population [11]. Other research, however, conclude that the incidence of depression increases with age, reaching the maximum frequency in late adulthood [12]. It should be emphasized that half or even more cases of geriatric major depression begin in late adulthood [7]. A comprehensive meta-analysis of 2011 indicates that in the over-75 age group, the symptoms of major depression are diagnosed in 4.6–9.3% of subjects. Taking into account also the subthreshold depressive symptoms, this result reaches the level between 4.5% and 37.4%. It should be emphasized that subthreshold depressive symptomatology is diagnosed two or even three times more often than the symptoms meeting the criteria for depression in the group over 55 years of age [13].

According to the latest WHO reports, depression is one of the most common mental and neurological disorders among older adults, affecting an estimated 7% of the population of people over the age of 60 [14]. A 2017 meta-analysis found that in most cultures depression is more common in women, especially in societies characterized by higher gender equality. This phenomenon implies indirectly that the background of depression is probably more biological than cultural. The traditional, culturally determined gender roles are likely to protect against depression by influencing the cultural meaning of existence. The results of these studies are contrary to the attitude of contemporary gender and feminist movements [15].

The studies published to date have demonstrated that the level of loneliness and the prevalence of depressive symptoms fluctuate throughout the life of an individual, with the correlation between loneliness and symptoms of depression maintaining a relatively constant level [16]. In the population of older adults, loneliness has been identified as a significant factor for the onset of depression symptoms [17]; however, an inverse correlation has also been indicated [18]. Demographic and psychosocial considerations are also important.

From another point of view, seniors are a sociologically heterogeneous group. An example of this is the lifestyle of seniors in the countryside and in large agglomerations [19]. In the countryside, the senior usually lives in a multigenerational family, having everyday contact with both close and more distant relatives [20]. On the other hand, the annual income of a senior in the Polish rural areas ranks on the borderline of poverty, and the social relations observed there are largely reminiscent of the amoral familism of Montegrano [19,21]. On the other hand, seniors in a big city usually live alone after losing their partner. They are isolated from family ties, and contacts with their children are usually limited to a few short meetings during the month. The city offers much better access to medical services than the rural areas, as well as a greater opportunity to participate in local and global culture [1,22,23].

The aim of this paper is to compare the perceived level of loneliness and depressiveness in seniors living in the country and in the cities and assess somatic morbidity and sociodemographic status as predictors of loneliness and depressiveness.

## 2. Materials and Methods

### 2.1. Study Design and Group

This was an observational, cross-sectional study. The study was conducted from 2019 to 2020 among ambulatory patients of four primary care units of the Public Healthcare Facility in Aleksandrów Łódzki, Poland.

The inclusion criteria were:

- informed consent for participation in the study,
- age of at least sixty.

The exclusion criteria comprised:

- lack of informed consent,
- diagnosis of major neurocognitive disorder,
- aphasia due to any reason,
- serious and unstable somatic disease,
- a severe psychological trauma within six months preceding the study,
- serious mental illness (e.g., schizophrenia or bipolar affective disorder or other psychotic disorder).

The study procedure involved completion of a set of self-reported questionnaires. Consecutive patients of the selected primary care units aged sixty or older were assessed regarding the exclusion criteria. Upon their routine ambulatory visit, they were given the questionnaires by the attending physician (who underwent a short training regarding the collection of the data). In total, 120 sets of questionnaires were distributed. *n* = 28 patients did not return the form, which was assumed as lack of informed consent. After signing the informed consent, the participants were asked to complete a set of questionnaires: authors’ survey on sociodemographic and clinical data and recognized psychometric tools (described below). Upon completion of the questionnaires and clinical data, the patients were once again verified regarding the exclusion/inclusion criteria. The final sample comprised *n* = 92 patients.

### 2.2. Operationalization of the Variables-Questionnaires

The severity of depressiveness was measured using the Beck Depression Inventory version II (BDI)adapted to Polish, validated and issued by the Psychological Test Laboratory of the Polish Psychological Association [24,25]. The questionnaire comprises 21 items considering occurrence of depressive symptoms in the past two weeks. Each item is scored from 0 to 3–the higher the score, the greater the severity of depressiveness [24,25].

The sense of loneliness was measured with the De Jong Gierveld Loneliness Scale (DJGLS) in a Polish adaptation by Paweł Grygiel et al. [26]. The tool consists of eleven items, of which five refer to the emotional dimension of the loneliness, and six—to the social aspect. Each item comprises five-point answer scale. An increase in the DJGLS total score suggest a rise in the sense of loneliness.

The demographic details were collected in the form of a diagnostic survey—a questionnaire of our own design. The questionnaire contains questions about age, gender, form of housing, marital status, number of children, the amount of pension or other benefit received, and the diseases. This questionnaire was completed with the help of an interviewer from the research team, trained to explain the individual questions to the respondents, and to structure the diseases that occur.

### 2.3. Ethical Considerations

The study was conducted according to the institutional and national ethical standards and in accordance with the guidelines of the Declaration of Helsinki. Ethical review and approval were waived for this study, due to observational character of the study and involvement of non-invasive measures (self-reported questionnaires).

### 2.4. Statistical Analysis

The statistical analysis was performed using STATISTICA 13.1 with medical add-on software. The categorical variables were presented as numbers with percentages. Pearson’s Chi^2^ test was used to assess the associations between variables in 2 × 2 contingencies. If the expected value in any of the 2 × 2 table cells was below 5, Fisher’s exact test was utilized instead. Mann–Whitney’s U test was used for contingencies greater than 2 × 2. The continuous variables were characterized by their minimum-to-maximum range, mean value, and standard deviation. The normality of the distribution of the variables was verified with Shapiro–Wilk test and visual analysis of the histograms. Central limit theorem was utilized. The heterogeneity of variance between the subgroups was checked with the Levene’s test. Intergroup comparisons were conducted by analysis of variance with Welch’s *t*-test, due to the lack of homogeneity of variance. The Pearson’s correlation quotient was calculated to assess the association between two continuous variables. Two linear regression models were constructed to predict the BDI score and DJGLS score. All variables of interest (characterized in Table 1) were considered as the potential predictors. The qualitative variables were coded with sigma-restrictions. Initially, all of the predictors were tested in single-predictor linear regression models. If the predictor variable was associated with the predicted variables with *p* < 0.1, it was included in the model. Both linear regression models were adjusted for the sex and age. An analysis of residuals was performed for each model to assess the validity of assumptions of normality, homoscedasticity, and independence between observations (with the Durbin–Watson test). The tolerance indices were analyzed to track possible multicollinearities. The effect sizes were assessed in two manners: for each model as a whole (coefficient of determination R^2^) and for each parameter in the model (semi- partial correlation sR). Those quotients may be interpreted in terms of Cohen’s thresholds for small (0.1), medium (0.3), and strong correlation (0.5). 10-fold cross-validation was performed for both of the models to assess their stability. In addition, post-hoc calculation of achieved power in the constructed regression models was performed with G*Power software. The level of significance was adopted for α = 0.05.

## 3. Results

### 3.1. Intergroup Comparisons by Place of Residence

There was a statistically significant difference in mean age between the older adults living in the city and in the rural area, with the former being on average older (F = 6.753, *p* < 0.05). Older adults living in the city had a higher monthly income compared to the residents of the rural areas (F = 23.481, *p* < 0.001). There was a significant difference regarding the structure of the number of children between the studied subgroups: almost 70% of the city residents had up to two children, while almost 50% of the older adults living in the country had at least three children (Z = −6.047, *p* < 0.001). Almost all of the rural area residents and 21% of the city residents lived in a house (Chi^2^ = 55.495, *p* < 0.001). There was a statistically significant relationship between the place of residence and the co-inhabitants; 34% of the city residents and 7% of the rural area residents declared that they lived alone. Interestingly, 44% of the rural area residents reported that they lived with their spouse and children, and a similar proportion (43%) of the city residents reported to live with their spouse alone (Z = −3.641, *p* < 0.001). City residents had on the average higher both BDI (F = 20.769, *p* < 0.001) and DJGLS scores (F = 26.360, *p* < 0.001). No other differences regarding the studied variables of interest were statistically significant, including the proportion of the reported morbidities. The detailed statistics are presented in Table 1.

### 3.2. Correlation between Severity of Depression and Loneliness

**Figure 1:**
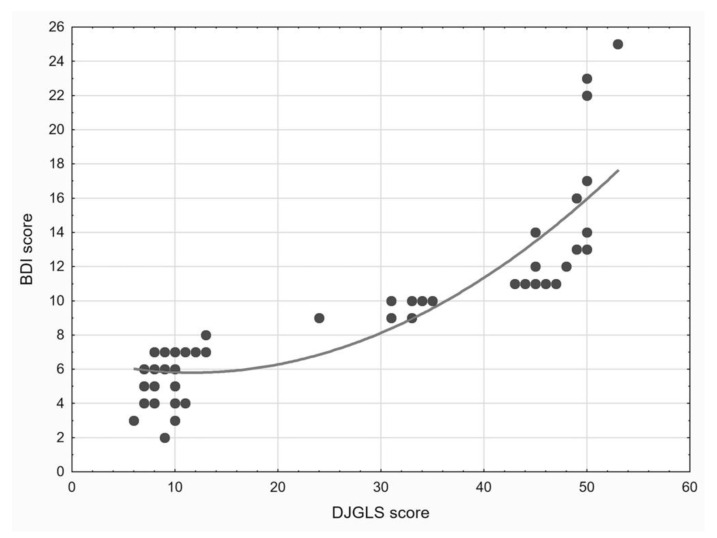
A scatter plot depicting the association between the De John Giervald Loneliness (DJGLS) scale score and Beck Depression Inventory-II (BDI) score in the studied group of the older adults living in the city and in the rural area. The curve indicates the modelled, non-linear relationship between the two scores.

There was a strong, positive, and statistically significant correlation between the BDI and DJGLS scores (R = 0.855, *p* < 0.001), with a strong size of the effect of the relationship (R^2^ = 0.731). On a scatter plot depicting that correlation, it can be noticed that the association between the two variables may be described more adequately by a polynomial function rather than a linear function (see Figure 1).

### 3.3. Prediction of the Severity of Loneliness

The constructed linear regression model predicting the DJGLS score had a high coefficient of determination and was adjusted to the empirical data (R^2^ = 0.856, F = 39.736, *df* = 14, *p* < 0.001). The above can be interpreted to mean that the predictor variables explained 86% of variance in the DJGLS score.

There was a statistically significant reduction in the cumulative correlation coefficient (and thus, coefficient of determination) for the model after ten-fold cross-validation: from R = 0.937 to R = 0.879. Nonetheless, the coefficient remained high, meaning that the model kept its predictive value (Table 2). The achieved post-hoc power of the model was 0.999.

### 3.4. Prediction of the Severity of Depression

The constructed linear regression model predicting the BDI score exhibited a high coefficient of determination and was adjusted to the empirical data (R^2^ = 0.842, F = 31.248, *df* = 16, *p* < 0.001). The above can be interpreted to mean that the predictor variables explained 84% of variance in the BDI score (Table 3).

There was a statistically significant reduction in the cumulative correlation coefficient (and coefficient of determination) for the model after ten-fold cross-validation: from R = 0.933 to R = 0.862. However, it should be noticed that the coefficient remained high, indicating that the model maintained its predictive value. The achieved post-hoc power of the model was 0.999.

## 4. Discussion

Preliminary results of an observational study into predictors of loneliness and depression among older patients are presented. In the model of prediction of the severity of loneliness, the relatively strongest effect was observed for those living alone vs. those living with the spouse. This result seems intuitively justified, as human society is organized in a “nest-like” way [27]. The nucleus of each nest is a strong dyadic bond between two people. For many people, a strong stress is associated with having no partner, and even greater stress with his/her loss [28] Seniors in a dyadic relationship are sooner or later at risk of losing their loved one. The bereavement caused by this fact, in the absence of support and the resources of coping with stress depleted with age, can lead to the development of a depressive episode or dysthymia [29].

The determination factor for the DJGLS and BDI prediction models was 86% and 87%, respectively—i.e., very high, especially for models containing psychometric variables. This work can be said to provide a relatively complete model explaining the depressiveness and loneliness of the older adults who are chronically ill. However, this statement must be cautious. The studied group is relatively small and certainly not representative of the entire senior population.

Our current results indicate that there might be a need to improve social support in the late adulthood as an intervention to diminish the sense of loneliness. Rondán-Cataluña et al. suggest that social networks can be an important way to improve seniors’ support networks [30]. Zheng and Chen presented interesting data on the senior support network in China. According to a study by Chinese researchers, as people get older, their family network expands, while the network of their friends shrinks. In addition, the scale of the expansion of the family network of older adults in rural areas is much greater than in the city, while the scale of the shrinkage of their network of friends is smaller compared to its urban counterpart. The impact of the family network on the well-being of seniors living in the country demonstrates a marked increase with age. However, there are no noticeable changes in urban groups of older adults of different ages [31].

While the difference in depressiveness and loneliness between city dwellers and villagers is evident (to the detriment of the former), regression models provide us with additional information. It turns out that the relationship of living in the village vs. in the city ceases to be statistically significant. However, the statistically significant predictors include: the number of children, living alone vs. living with the spouse only, as well as living with children and the spouse vs. living only with children. It can therefore be concluded that lower rates of depressiveness and loneliness among older adults living in the country than among those living in the cities are due to the maintained support network and not directly to rural living itself. This seems to be in line with previous publications [31]. According to Australian research, the most important predictor of good quality of life and lack of depression in seniors is the social support network, which is in line with the results of our research and the research conducted by Chinese scientists.

The predictors of DJGLS and BDI scores delineated in the constructed models are for the most part common in this work. Once again, a strong correlation between depressiveness and loneliness in the population of seniors with somatic illnesses treated in primary care needs to be emphasized [32].

The shortcoming of our work is the lack of randomity of the study group selection. The correlations between the variables would possibly have had a smaller value if the group had been randomly selected. The study group was not very large, so the result obtained for large populations may begin to differ from the result obtained by us. Studies on a large population should be conducted on a multicenter basis rather than based on two locations, as the result we obtained may have been influenced by the specificity of the local community, which is true predominantly for the rural areas.

The obtained results apply only to people of senior age who actively benefit from the medical services of a primary care physician. Therefore, the sample is not representative of the entire senior population, consisting of both healthy and sick people. On the other hand, it should be noted, that there are very few healthy seniors without any diseases [33]. The answer to the question we asked would be provided by a survey of a random sample of the senior population and by determining the percentage of seniors who receive primary healthcare regularly. Due to the fact that these people suffer from somatic diseases, the results of certain scales may be overestimated relative to the general population. For instance, the correlation we have obtained between BDI and DJGLS results is very high. The force of the effect for this dependence is also high, even compared to the sample from the Polish DJGLS test standardization [26]. Our result probably indicates a strong association of depressive symptoms in the surveyed seniors with the sense of loneliness they feel. For other population groups, for example, young people, this correlation does not have to be so significant. Depression often also affects people in relationships, and its background is often associated with affective disorders (single and bipolar) [34]. Possibly, the depression we observed in seniors is a reactive syndrome caused by loneliness.

There are also a number of variables demonstrated by empirical studies to be risk factors for loneliness and depression that have not been taken into account in this study. The examples include urinary incontinence in women, as well as some macro- and microeconomic factors [18]. The population investigated by us included subjects with somatic diseases. With a larger study population, an analysis of the impact of individual diseases on the mental state and loneliness in seniors should be performed. It is known that the sense of loneliness in seniors depends on the presence of inflammatory diseases and correlates with serous concentrations of interleukin-6 [35]. Another somatic risk factor for loneliness is the presence of a malignant tumor [36]. This appears to be congruent with our study, since history of a neoplasm predicted rise in both depression and loneliness. Moreover, it should be remembered that the incidence of tumors increases with age [36]. Hearing impairment associated with old age is also a risk factor for mood disorders in seniors [37].

It would also be necessary to consider whether loneliness is the cause of depression or its effect, or whether both variables depend on common factors and affect each other in a circular way. Many authors suggest that loneliness is a risk factor for depression in seniors [38,39,40,41,42]. However, this thesis requires the selection of methodology, statistical models and tools which would determine the nature and the cause-and effect relationship of this relationship.

## 5. Conclusions

A strong relationship between loneliness and depressiveness was found among older adults. Higher indices were seen among those living in the city than those living in the rural area. Sociodemographic status and somatic morbidities accounted for around 90% of variance of depressiveness and loneliness scores in the studied group. Living alone was found to be the strongest relative predictor of both loneliness and depressiveness in the studied sample of the patients in the late adulthood. Our current results suggest that there might be a need to improve social support for the older adults as an intervention to diminish the sense of loneliness and depression.

## Figures and Tables

**Table 1:** Characteristics of the studied group of older adults, with a comparison between groups delineated by the place of residence (city or rural area).

	City (*n* = 47)	Rural Area (*n* = 45)
Age, M ± SD (min-max) *	68.9 ± 4.6 (65–85)	71.6 ± 5.5 (64–85)
Female sex, *n* (%)	27 (57%)	32 (71%)
Current income monthly in PLN, M ± SD (min-max) **	1773.0 ± 323.3 (1280–2700)	1505.4 ± 192.6 (1200–1950)
Number of children, *n* (%): **		
none	10 (21%)	0 (0%)
one	22 (47%)	4 (9%)
two	12 (26%)	21 (29%)
three	3 (6%)	13 (29%)
four	0 (0%)	5 (11%)
five	0 (0%)	2 (4%)
Marital status, *n* (%):		
single	5 (11%)	0 (0%)
married	25 (53%)	32 (71%)
widowed	7 (15%)	10 (22%)
divorced	10 (21%)	3 (7%)
Informal relationship, *n* (%)	6 (13%)	2 (4%)
Place of habitat, *n* (%): **		
house	10 (21%)	44 (98%)
flat	37 (79%)	1 (2%)
Co-inhabitants, *n* (%): **		
lives alone	13 (34%)	3 (7%)
with spouse only	20 (43%)	12 (27%)
with children only	1 (2%)	8 (18%)
with spouse and children	5 (11%)	20 (44%)
with other close person	5 (11%)	2 (4%)
Morbidities, *n* (%):		
Type 2 diabetes	12 (26%)	14 (31%)
Arterial hypertension	12 (25%)	8 (18%)
Heart failure	9 (19%)	7 (16%)
Upper gastrointestinal tract disease	3 (6%)	5 (11%)
Arthritis (rheumathoid or ostheoarthritis)	2 (4%)	3 (7%)
Asthma	1 (2%)	1 (2%)
Stroke in the past	1 (2%)	1 (2%)
Visual impairment (e.g., cataract)	2 (4%)	4 (9%)
Neoplasm	2 (4%)	0 (0%)
Other	10 (21%)	4 (9%)
BDI score, M ± SD (min-max) **	9.6 ± 4.9 (4–25)	6.1 ± 1.9 (2–11)
DJGS score, M ± SD (min-max) **	26.4 ± 17.6 (8–53)	11.4 ± 9.1 (6–46)

*n*: number of observations, M: mean, SD: standard deviation, Min: minimum value, Max: maximum value, PLN: Polish New Zloty (currency), BDI: Beck Depression Inventory II, DJGLS: De Jong Gierveld Loneliness Scale; markings of statistically significant differences: * *p* < 0.05, ** *p* < 0.001.

**Table 2:** Parameters of the linear regression model predicting the De Jong Gierveld Loneliness Scale score in the studied group of the older adults living in the city and in the rural area.

		B	B 95%CI		sR
Intercept		27.799 *	3.008	52.591	
Age		−0.034	−0.366	0.299	−0.008
Female sex		1.750	−0.005	3.505	0.079
Number of children		−2.826 **	−4.604	−1.048	−0.126
Marital status	single vs. married	−2.227	−8.639	4.185	−0.027
	widowed vs. married	−2.120	−8.766	4.525	−0.025
	divorced vs. married	−4.377 *	−7.740	−1.014	−0.103
Place of residence-city vs. rural area		1.819	−0.640	4.277	0.059
Place of habitat-flat vs. house		−2.255	−4.671	0.161	−0.074
Co-inhabitants	alone vs. with spouse	29.727 †	22.344	37.111	0.319
	with children only vs. with spouse	−1.505	−11.709	8.699	−0.012
	with spouse and children vs. with spouse	−15.157 †	−23.453	−6.861	−0.145
Monthly income (pension)		0.004	−0.003	0.010	0.045
Visual impairment		−1.208	−4.312	1.896	−0.031
Neoplasm		11.390†	6.441	16.339	0.182

B: unstandardized parameter, CI: confidence interval, sR: semipartial correlation (size of effect), markings of statistically significant effects: * *p* < 0.05, ** *p* < 0.01, † *p* < 0.001.

**Table 3:** Parameters of the linear regression model predicting the Beck Depression Inventory II score in the studied group of the older adults living in the city and in the rural area.

		B	B 95%CI		sR
Intercept		9.916 **	2.902	16.930	
Age		0.071	−0.021	0.164	0.064
Female sex		0.529 *	0.055	1.004	0.093
Number of children		−0.874 **	−1.355	−0.393	−0.151
Marital status	single vs. married	0.318	−1.475	2.112	0.015
	widowed vs. married	−0.745	−2.550	1.061	−0.034
	divorced vs. married	−0.686	−1.610	0.238	−0.062
Place of residence-city vs. rural area		0.010	−0.659	0.679	0.001
Place of habitat-flat vs. house		−0.007	−0.661	0.647	−0.001
Co-inhabitants	alone vs. with spouse	4.823 †	2.826	6.820	0.201
	with children only vs. with spouse	−0.007	−2.779	2.764	0.000
	with spouse and children vs. with spouse	−2.490 *	−4.734	−0.246	−0.092
Monthly income		0.002	0.000	0.003	0.075
Asthma		2.049 **	0.774	3.325	0.134
Diabetes mellitus		0.167	−0.278	0.613	0.031
Visual impairment		−0.315	−1.178	0.547	−0.030
Neoplasm		6.769 †	5.406	8.132	0.413

B: unstandardized parameter, CI: confidence interval, sR: semipartial correlation (size of effect), markings of statistically significant effects: * *p* < 0.05, ** *p* < 0.01, † *p* < 0.001.

## Data Availability

The data presented in this study are available on request from the corresponding author.
